# Oocyte biology: at the center of assisted reproduction technologies

**DOI:** 10.1093/biolre/ioac017

**Published:** 2022-02-01

**Authors:** Rebecca L Robker

**Affiliations:** Robinson Research Institute, School of Biomedicine, The University of Adelaide, Adelaide, South Australia, Australia; Biomedicine Discovery Institute, Department of Anatomy and Developmental Biology, Monash University, Clayton, Victoria, Australia

##  

Medicine has been transformed by advances in assisted reproductive technologies (ART). The advent of in vitro fertilization in the 1970s has given the estimated one in six couples affected by infertility an opportunity to have their own children. In parallel, the development of protocols to store gametes and mature them later, in vitro, enables reproduction to be postponed. Better understanding of how obesity, aging, and chemotherapies affect female fertility has raised awareness and facilitated the development of strategies to mitigate the detrimental impacts. Biomarker identification and innovative detection modalities allow measurement of both high-quality and poor-quality gametes, increasing ART success rates. At the heart of these extraordinary advances has been discovery research in oocyte biology. This Special Issue of Biology of Reproduction details our current understanding of oocyte biology and the scientific research that has revolutionized reproductive medicine.

## Advances in our fundamental understanding of oocytes and developmental biology

The oocyte is a remarkable single cell that grows and matures within the ovary, and following fertilization, provides the molecular building blocks to create an embryo capable of implantation and generation of a new individual. Oocytes that are immature (i.e., at the germinal vesicle stage) do not have full developmental competence to produce an embryo; an appropriate maturation signal is essential for conception to occur either in vivo or in vitro. Marc-Andre Sirard likens the events of folliculogenesis and maturation to the ripening of fruit, with the ovarian follicle holding its precious seed, the oocyte. In vivo, oocyte maturation occurs in response to the ovulatory luteinizing hormone surge, which triggers dramatic cytoplasmic and nuclear events. Of particular importance, Karen Schindler describes the intricate molecular processes that control formation of the metaphase II (MII) spindle—information that is essential for understanding aneuploidy.

Once progression to MII is complete, oocyte activation can be initiated by sperm. The fertilization event commences the transition to zygote and a profound process of reprogramming to pluripotency. These precisely regulated mechanisms essential for conception are described by Kiho Lee and colleagues. They also summarize artificial oocyte activation techniques that are used clinically to increase fertilization rates and improve embryo quality as well as to generate somatic cell nuclear transfer embryos for production of genetically engineered animals and to induce parthenogenesis for studies of early development and stem cell biology.

The identification of these key events in oocyte growth, maturation, meiotic progression and activation, as well as the somatic cell regulatory factors, has enabled the development of technologies that can detect oocyte quality. The team of Gary Smith summarizes a myriad of diagnostic approaches that involve assessments of the oocyte environment, as well as new techniques, particularly imaging modalities, that are on the horizon and able to assess the oocyte directly.

## Major new areas of future investigation

This Special Issue also highlights key research areas in oocyte biology which are being rapidly translated to new advances in reproductive medicine. In parallel, these basic science discoveries in animal models are being developed into technologies to improve animal production, pest control, and species conservation. One of the most compelling areas is in the rapidly advancing field of in vitro follicle development. Jing Xu provides a detailed history of the development of in vitro follicle culture, including protocols that have improved oocyte growth and development. This technology, which is increasingly being used in a number of species, will dramatically extend our capacity to generate mature oocytes from progressively more immature primordial cells.

A second major area of future investigation is in oocyte vitrification technologies where increased efficiency is needed to improve pregnancy rates from frozen oocytes. Jeremy Chang and co-authors describe the diverse array of cellular damage that can occur with current oocyte freezing protocols due to both the effects of chilling and/or cryoprotectant toxicity. They also summarize the clinical trials that have evaluated pregnancy outcomes from vitrified compared to fresh oocytes. Oocyte vitrification is increasingly being used, for instance, for fertility preservation for cancer patients and for social reasons, and thus ongoing work to optimize the technology will help guarantee healthy pregnancies.

A third major advancement is in the clinical adoption of oocyte in vitro maturation (or IVM). Rebecca Krisher summarizes the current state-of-the-art IVM as used in the treatment of human infertility and fertility preservation, its advantages and shortcomings, and examines what is next for this technology. Even though IVM is no longer considered experimental, its use in routine IVF is still limited. However, with continued improvements, IVM could become a feasible alternative to conventional ovarian stimulation, offering a more patient-friendly and cost-effective option and eliminating the incidence of ovarian hyperstimulation syndrome. Toward these goals, Robert Gilchrist highlights his team’s development of pre-IVM oocyte maturation systems that involve modulating cGMP and/or cAMP to artificially maintain meiotic arrest prior to IVM. Such pre-IVM approaches are proving useful in both medical and veterinary assisted reproduction.

## Restoring oocyte quality to improve female fertility and offspring health

There are a number of physiological contexts where oocyte quality is poor, leading to sub-fertility and non-viable pregnancies. One of the most pervasive and intractable issues in reproductive medicine is the decline of ovarian function with age—both oocyte number and quality are dramatically impacted by aging. As the average age of childbearing has increased over the last two decades, the number of ART cycles in women aged ≥38 has doubled [1]. Female aging causes very specific effects on oocytes, particularly changes to mitochondrial activity and spindle formation, that are potentially linked to fibrosis and inflammatory changes in the ovarian stromal compartment. To investigate this, Francesca Duncan provides a comprehensive summary of the changes that occur in the ovarian microenvironment of women in response to aging. These include genetic, epigenetic, transcriptomic, proteomic, and metabolomic alterations in granulosa and cumulus cells and follicular fluid as well as effects on inflammatory profile, redox status, and extracellular matrix.

Obesity also impairs female fertility, and the clinical effects of this are expected to rise due to its prevalence: in the USA, the average young adult (18–25 years old) is now overweight or obese [2]. As summarized by Macarena Gonzalez, obesity impacts each step of a woman’s ART journey from poor gonadotropin response to fewer cumulus-oocyte complexes collected at aspiration, a greater proportion of immature oocytes, and following embryo transfer, increased risks of failed implantation and miscarriage. Further, accumulating evidence indicates that obesity in mothers influences obesity susceptibility in offspring via signals contained in the oocyte. Thus, identifying the nature of these factors will not only assist women with metabolic disease to reach their fertility goals but will also help break the cycle of intergenerational obesity.

Exogenous insults also affect oocyte development and viability. For instance, several commonly used chemo- and radiotherapies are toxic to oocytes. Advances in oncology diagnostics and treatments enable many young female cancer patients to survive and live healthy lives. As such, the multi-disciplinary field of “oncofertility” has arisen to better understand and mitigate the detrimental impacts of anti-cancer therapies on fertility and to provide cancer patients with fertility preservation options. Shuo Xiao summarizes the cellular mechanisms by which different types of cancer treatments impair female fertility and provides a detailed overview of the currently available technologies and guidelines for human fertility preservation. His review also highlights emerging oncofertility technologies and knowledge gaps where more research is needed to demonstrate the efficacy and safety for long-term child health outcomes. As success rates for these techniques rapidly improve, they are transforming the lives of female cancer survivors.

The multi-faceted roles of mitochondria in oocyte biology are described by Deepak Adhikari and colleagues, including newly emerging functions in epigenetic regulation. There is current focused attention on this biology as mitochondrial donation techniques that generate embryos from oocytes of two women are being developed and used clinically in some countries. Oocyte mitochondria are known to be damaged in many contexts, for instance, with reproductive aging and obesity; and their review highlights pharmaceuticals that improve mitochondrial function and therefore provide potential therapeutics to improve female fertility.

Cumulatively, the review articles in this Special Issue highlight several key themes. First, even in the context of ART, oocyte quality is a rate-limiting factor for female fertility, and molecular events at conception influence embryo development and offspring health. Second, the ovarian environment is critical for normal oocyte maturation and as such provides biomarkers for assessment of gamete quality. Lastly, basic science discovery in animal models paves the way for clinical advances and improved ART, enabling possibilities for fertility preservation, options for delaying childbearing, and therapies that facilitate a healthy conception and pregnancy. I would like to thank the authors for their outstanding reviews which, combined, clearly demonstrate that fundamental discoveries in oocyte biology are indeed at the heart of many current and future technologies that are transforming reproductive medicine.
